# Health disparity and healthcare utilization inequity among older adults living in poverty in South Korea: a cross-sectional study

**DOI:** 10.1186/s12877-022-03686-0

**Published:** 2022-12-27

**Authors:** Ah-Young Kim, Moon Sil Seo, Hye-Young Kang

**Affiliations:** 1 Department of Pharmaceutical Medicine and Regulatory Sciences, College of Medicine, College of Pharmacy, Yonsei University, Seoul, South Korea; 2 College of Pharmacy, Yonsei Institute of Pharmaceutical Sciences, Yonsei University, 85 Songdogwahak-ro, Incheon , 21983 Incheon, South Korea; 3 Graduate Program of Industrial Pharmaceutical Science, Yonsei University, Incheon, South Korea

**Keywords:** Economic status, Health disparity, Geriatric disease, Medical aid, National Health Insurance

## Abstract

**Background:**

Korea has a two-tiered universal health security system: the wage-based National Health Insurance (NHI) program and government-subsidized Medical Aid (MA) program. Beneficiaries of the MA program belong to the lowest economic class. This study aims to investigate the association between economic status—defined as NHI or MA enrollment—and health disparity of older people aged ≥ 65 years in South Korea.

**Methods:**

The claims records of 672,525 older age population from the 2017 Health Insurance Review and Assessment Service-Adult Patient Sample were used to estimate adjusted odds ratios (aORs) of MA vs. NHI beneficiaries for prevalence for common geriatric diseases. Logistic regression and negative binomial regression were used to investigate the association between economic status and prevalence or healthcare utilization for each disease.

**Results:**

MA beneficiaries showed significantly higher prevalence than NHI beneficiaries for seven out of nine diseases (aORs ranging from 1.18 to 1.95). The discrepancy in the prevalence between the two groups was highest among those aged 65–69 years (aORs: 1.34–2.94), and diminished as they got older (aORs: 1.05–1.67). MA beneficiaries had significantly more outpatient visits to treat six diseases (aORs: 1.07–1.28), and more hospitalization to treat seven diseases (aORs:1.08–1.73) than NHI beneficiaries.

**Conclusion:**

The higher prevalence of common geriatric diseases among MA than NHI beneficiaries confirms unfavorable health disparity in the elderly living in extreme poverty. Similar or higher healthcare utilization in treating the same conditions among MA beneficiaries suggests a low possibility of inequity for access to healthcare resources covered by the universal health security system due to poor economic status. Greater excess use of inpatient than outpatient care by MA beneficiaries implies that the condition of poor older adults might be more severe when diagnosed with the same disease.

## Background

Population ageing is a worldwide phenomenon with prominent impact on the global burden of disease and public health. Since 2017, South Korea became an aged society as older people aged 65 years or more accounted for more than 14% of the population [1]. An aging society is defined as 7% or more of the population aged 65 or older, 14% or more as an aged society, and 20% or more as a super-aged society [2]. As of 2021, people aged 65 years or more make up 16.8% of the population. With rapid population aging, the proportion of older adult population is projected to be more than 40% of the population in 2050 in South Korea. Korea is expected to rank first among OECD countries in 2050 as a country with the fastest growing older adult population [3, 4]. Poverty in old age is a common challenge of an aged society. Among Organization for Economic Co-operation and Development (OECD) countries, South Korea has the highest poverty rate (proportion of households with income less than 50% of the median household disposable income) among older people (49.6%), which is over four times higher than the OECD average of 12.6% [3, 5].

Korea has a two-tiered universal health security system: the National Health Insurance (NHI) program is a wage-based, contributory insurance program; and the Medical Aid (MA) program is a government-subsidized public assistance program for poor and medically indigent individuals [6]. Beneficiaries of the MA program belong to the lowest economic class in Korea, and primarily live on financial aid from the national social security fund and without family members working in the labour market. While MA beneficiaries compose 3% of the population, older people comprise 36.0% of the MA population. [7], which indicates significant poverty among the older population in Korea.

People living in poverty have limited access to healthcare services, which may delay detection of diseases, and increase the risk of disease progression, acute exacerbations, and death [8]. This phenomenon may be even worse among the older adults, who have more vulnerable health profiles than the general population, causing severe health inequality problem due to economic status. The inverse association between economic status and the impaired health function in old age is observed in conditions such as chronic obstructive pulmonary disease (COPD), cognitive function, fractures, and Parkinson’s disease [9–12]. However, these studies examined old-age health disparity by economic status in a single condition; moreover, they did not clarify whether the health disparity stays consistent across the wide range of conditions or varies according to the disease.

Therefore, we conducted this study to compare the prevalence of common geriatric diseases between NHI and MA beneficiaries aged 65 years or older to examine differences in the magnitude of health disparity across geriatric diseases between older adults living in extreme poverty and other older adults. Furthermore, we assessed disparity in healthcare utilization provided to treat the same disease in the population between these two groups. Among various geriatric diseases, we selected nine diseases for the analysis because they commonly cause high mortality, physical disability, poor quality of life, and increased medical expenses in the population aged 65 years or older [13]: depression, hip fracture, chronic heart failure (CHF), COPD, cerebrovascular accident (CVA), Parkinson’s disease (PD), acute myocardial infarction (AMI), transient ischemic attack (TIA), and lung cancer. We hypothesized that being female, being older, living in non-city areas, and having a higher CCI score were positively associated with the probability of having the disease.

## Methods

### Data sources and study population

This study was conducted using the 2017 Health Insurance Review and Assessment Service-Aged Patient Sample data (HIRA-APS, serial number: HIRA-APS-2017-0020). They are national representative cross-sectional claim records with a 10% random sample of population aged ≥ 65 years old (approximately 700,000) who utilized NHI-covered healthcare services in 2017. The data include anonymized patient identification number with sex, age, and information about NHI-covered healthcare utilization including diagnosis, procedures, prescribed drugs, and reimbursed costs. Study participants were defined as patients aged ≥ 65 years whose beneficiary status of NHI or MA had not changed during the year. The nine geriatric diseases were identified using International Classification of Diseases (ICD)-10 diagnostic codes: depression (ICD-10 code: F31-F34, F41.2), hip fracture (S72), CHF (I50), COPD (J43, J44), CVA (I60-I69), PD (G20), AMI (I21, I22), TIA (G45), and lung cancer (C33, C34).

### Statistical analysis

For each of the nine conditions, age-specific prevalence rates per 10,000 people aged 65 years or older were calculated for NHI and MA beneficiaries, respectively, and presented in three age groups: 65–69, 70–74, and 75 years old or above.

Prevalence rate_i_ = (Number of NHI or MA beneficiaries having at least one hospitalization or two outpatient visits with a primary diagnosis of disease of interest_i_ / Total number of NHI or MA beneficiaries_i_) x 10,000.

Where, i = 1 if 65–69 years old, 2 if 70–74 years old, 3 if ≥ 75 years old.

Healthcare utilization associated with each condition was analyzed using claim records for healthcare services with the same diagnosis codes used to define the patient. The annual average number of outpatient visits and hospital admissions per patient with each condition was calculated. Logistic regression analysis was performed for each of the nine conditions to investigate the association between economic status (defined as either MA or NHI enrollment) and the prevalence of that specific condition. Covariates to control for confounding effect on the probability of having each condition included age, sex, residential area, and Charlson Comorbidity Index (CCI). If a patient was identified as living in the capital city or other metropolitan city, their residential area was classified as “city,” and “non-city areas” otherwise. CCI score is widely used to reflect individual’s baseline condition, with a higher score indicating a worse health status [14]. To evaluate the underlying severity of the patients’ condition owing to other than nine conditions, we calculated separate CCI scores for each of the nine logistic regression models excluding the condition of the dependent variable.

For the number of outpatient visits and hospitalizations per patient, negative binomial regression model was used, because the dependent variables were count variables. The distribution of the variables has a larger variance than the means; therefore, Poisson regression or negative binomial regression should be used. The Lagrange multiplier test was performed to confirm the over-dispersion, and it was confirmed that the *p*-value of the over-dispersion factor alpha was < 0.0001 [15].

All statistical analyses were conducted with SAS (Statistical Analytic Software) version 9.4., and were considered significant when *p*-value exceeded 0.05.

## Results

Among the nine diseases, CVA showed the highest prevalence for both the NHI (868.5 per 10,000 beneficiaries) and MA beneficiaries (1,197.1 per 10,000) aged 65 or above, followed by depression (NHI: 295.1, MA: 481.3), COPD (NHI: 161.6, MA: 284.0), CHF (NHI: 140.1, MA: 251.5), PD (NHI: 122.3, MA: 172.7), hip fracture (NHI: 82.4, MA: 151.4), AMI (NHI: 69.1, MA: 85.7), TIA (NHI: 68.2, MA: 80.7), and lung cancer (NHI: 74.3, MA: 73.5) (Table 1). Overall, the prevalence of each disease increased with age regardless of the type of beneficiaries, except for depression and lung cancer. For depression, prevalence decreased with age for MA beneficiaries, whereas the opposite trend was observed in NHI beneficiaries. For both beneficiary groups, prevalence of lung cancer increased up to 70–74 years of age, and decreased thereafter (Table 1).

**Table 1:** Age-specific annual prevalence rates of common geriatric diseases among Korean older adults

		Age (years)			
		Overall (≥ 65)	65–69	70–74	≥ 75
**Number (%) of beneficiaries from 2017 HIRA-APS data**					
	NHI	626,672 (93.2)	206,867 (95.5)	159,771 (93.9)	260,034 (91.0)
	MA	45,853 (6.8)	9,825 (4.5)	10,370 (6.1)	25,658 (9.0)
**Prevalence rate (per 10,000 beneficiaries)**					
CVA (ICD-10 code: I60-I69)	NHI	868.5	600.2	833.3	1103.7
	MA	1197.1	1058.5	1212.2	1244.1
	Crude OR (95% CI)	1.38 (1.34, 1.42)	1.76 (1.65, 1.89)	1.45 (1.37, 1.55)	1.13 (1.08, 1.17)
CHF (I50)	NHI	140.1	55.3	99.4	232.7
	MA	251.5	121.1	171.6	333.6
	Crude OR (95% CI)	1.79 (1.69, 1.91)	2.19 (1.81, 2.65)	1.73 (1.48, 2.02)	1.43 (1.33, 1.54)
TIA (G45, H340)	NHI	68.2	55.5	68.6	78.0
	MA	80.7	64.1	75.2	89.3
	Crude OR (95% CI)	1.18 (1.06, 1.32)	1.15 (0.90, 1.49)	1.10 (0.87, 1.38)	1.14 (1.00, 1.31)
AMI (I21, I22)	NHI	69.1	56.4	61.6	83.7
	MA	85.7	79.4	89.7	86.5
	Crude OR (95% CI)	1.24 (1.12, 1.38)	1.41 (1.12, 1.77)	1.46 (1.18, 1.80)	1.03 (0.90, 1.19)
COPD (J43, J44)	NHI	161.6	99.6	153.2	216.1
	MA	284.0	281.9	274.8	288.4
	Crude OR (95% CI)	1.76 (1.66, 1.86)	2.83 (2.49, 3.21)	1.79 (1.58, 2.03)	1.33 (1.24, 1.44)
Lung Cancer (C33, C34)	NHI	74.3	61.6	81.9	79.6
	MA	73.5	75.3	84.9	68.2
	Crude OR (95% CI)	0.99 (0.89, 1.11)	1.22 (0.97, 1.55)	1.04 (0.83, 1.29)	0.86 (0.73, 1.00)
Hip fracture (S72)	NHI	82.4	21.9	47.7	151.9
	MA	151.4	72.3	79.1	210.9
	Crude OR (95% CI)	1.84 (1.70, 1.99)	3.29 (2.56, 4.23)	1.66 (1.32, 2.08)	1.39 (1.27, 1.52)
PD (G20)	NHI	122.3	52.8	103.9	188.9
	MA	172.7	117.0	139.8	207.3
	Crude OR (95% CI)	1.41 (1.31, 1.52)	2.12 (1.83, 2.69)	1.35 (1.13, 1.60)	1.10 (1.00, 1.20)
Depression (F31- F34)	NHI	295.1	243.9	302.2	331.5
	MA	481.3	585.2	557.4	410.8
	Crude OR (95% CI)	1.63 (1.56, 1.71)	2.40 (2.20, 2.62)	1.84 (1.69, 2.02)	1.24 (1.16, 1.32)

*AMI* acute myocardial infarction, *CHF* congestive heart failure, *CI* confidence interval, *COPD* chronic obstructive pulmonary disease, *CVA* cerebrovascular accident, *HIRA-APS* Health Insurance Review and Assessment Services-Aged Patient Sample, *ICD* International Classification of Diseases, *MA* Medical Aid, *NHI* National Health Insurance, *OR* odds ratio comparing MA vs. NHI, *PD* Parkinson’s disease, *TIA* transient ischemic attack

**Fig. 1:**
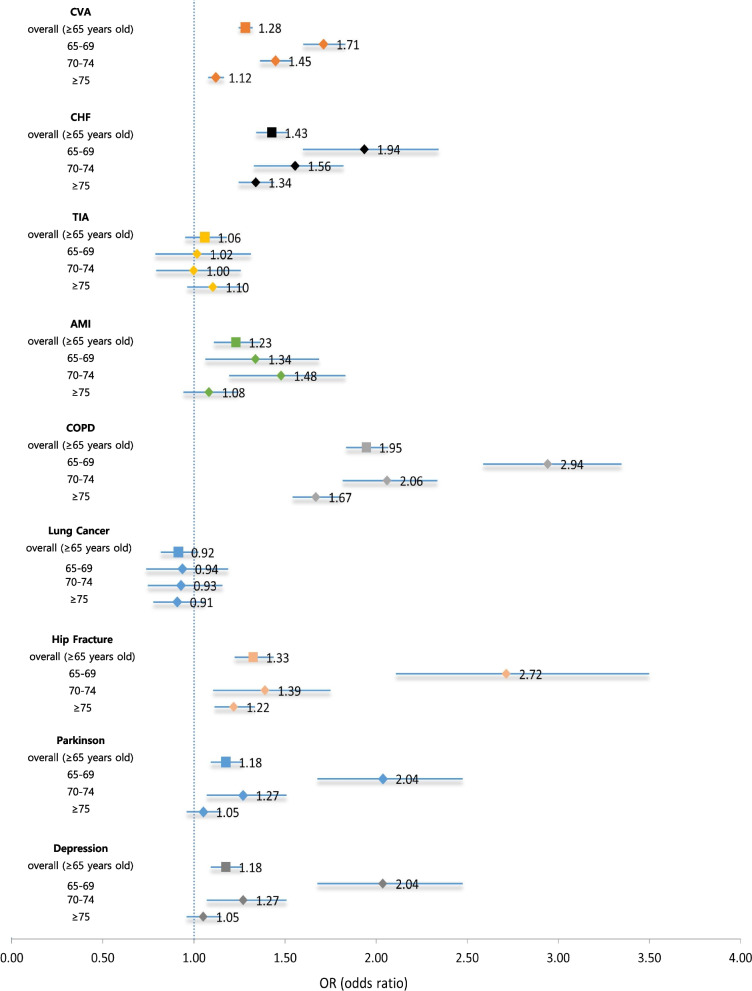
Adjusted ORs for prevalence of common geriatric diseases: Beneficiaries of MA vs. NHI. *AMI: acute myocardial infarction; CHF: chronic heart failure; COPD: chronic obstructive lung disease; CVA: cardiovascular accident; OR: odds ratio; MA: medical aid; NHI: national health insurance; TIA: transient ischemic attack. *Odds ratios were adjusted for age, sex, comorbidity, and area of residence using logistic regression models for each disease

MA beneficiaries showed significantly higher prevalence than NHI beneficiaries for eight geriatric diseases, excluding lung cancer, with significant crude odds ratios (ORs) ranging from 1.17 (for TIA) to 1.84 (for hip fracture) (Table 1). For all nine diseases, the discrepancy in the risk of getting the diseases between the two beneficiary groups was the highest in group aged 65–69 years, and diminished as they got older. After adjusting for age, sex, comorbidity, and area of residence in multivariate analysis, MA beneficiaries still showed significantly higher prevalence than NHI beneficiaries for seven conditions except for lung cancer and TIA (Fig. 1). The trend of greater ORs in the group aged 65–69 years old than the group aged 75 years or above remained the same. For example, MA beneficiaries showed about 1.34–2.94 times significantly higher risk of having AMI (adjusted OR: 1.34), CVA (1.71), CHF (1.94), depression (2.04), PD (2.04), hip fracture (2.72), and COPD (2.94) than NHI beneficiaries when they were 65–69 years old. When aged 75 years or above, the adjusted ORs were no longer significant for AMI, PD, and depression. Only four diseases showed a significantly higher chance of the disease among MA than NHI beneficiaries: CVA (adjusted OR: 1.12), hip fracture (1.22), CHF (1.34), and COPD (1.67).

**Fig. 2:**
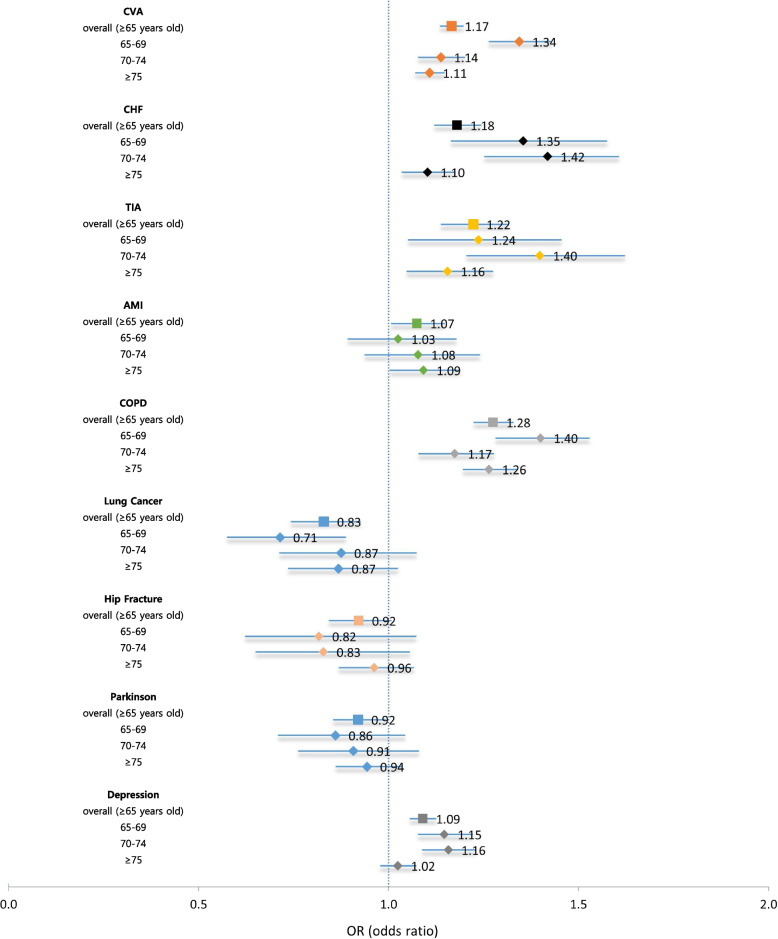
Adjusted ORs for the number of outpatient visits of common geriatric diseases: MA vs. NHI. AMI: acute myocardial infarction; CHF: chronic heart failure; COPD: chronic obstructive lung disease; CVA: cardiovascular accident; OR: odds ratio; MA: medical aid; NHI: national health insurance; TIA: transient ischemic attack. *Odds ratio were adjusted for age, sex, comorbidity, and area of residence using negative binomial regression models for each disease

The extent of healthcare utilization between NHI and MA older adults in treating the same disease was compared. Among the nine diseases, both NHI and MA groups had the most frequent outpatient visits in treating lung cancer (NHI: 10.27 visits, MA: 7.64 visits), depression (NHI: 8.98, MA: 9.49), PD (NHI: 6.81, MA: 5.27), and COPD (NHI: 5.32, MA: 6.30) (Table 2). According to the multivariate analysis, lung cancer, hip fracture, and PD showed no significant difference in the average annual number of outpatient visits per patient to treat the diseases between the two groups. MA patients with CVA (adjusted OR: 1.17), CHF (1.18), TIA (1.22), AMI (1.07), COPD (1.28), or depression (1.09) had significantly more frequent outpatient visits than NHI patients (Fig. 2). Only lung cancer, especially in those aged 65–69 years, showed significantly fewer outpatient visits among MA than NHI patients (adjusted OR: 0.71).

**Table 2:** Healthcare utilization for common geriatric diseases among Korean older adults

	Average number of outpatient visits per patient			Average number of hospital admissions per patient		
	NHI	MA	Ratio of MA to NHI (95% CI)	NHI	MA	Ratio of MA to NHI (95% CI)
CVA	4.72	5.12	1.08 (1.05, 1.12)	0.35	0.50	1.41 (1.35, 1.48)
CHF	3.37	3.86	1.15 (1.07, 1.23)	0.31	0.41	1.31 (1.17, 1.47)
TIA	4.49	4.90	1.09 (0.97, 1.23)	0.20	0.30	1.49 (1.19, 1.86)
AMI	3.62	3.65	1.01 (0.90, 1.13)	0.37	0.44	1.18 (0.98, 1.42)
COPD	5.32	6.30	1.18 (1.11, 1.26)	0.27	0.45	1.67 (1.50, 1.86)
Lung cancer	10.27	7.64	0.74 (0.66, 0.84)	1.46	1.60	1.10 (0.95, 1.26)
Hip fracture	2.63	2.35	0.89 (0.81, 0.98)	1.25	1.35	1.09 (0.98, 1.21)
PD	6.81	5.27	0.77 (0.72, 0.84)	0.52	0.70	1.34 (1.20, 1.50)
Depression	8.98	9.49	1.06 (1.01, 1.11)	0.04	0.08	1.78 (1.50, 2.12)

*AMI* acute myocardial infarction, *CHF* congestive heart failure, *CI* confidence interval, *COPD* chronic obstructive pulmonary disease, *CVA* cerebrovascular accident, *MA*: medical aid, *NHI* national health insurance, *PD* parkinson’s disease, *TIA*: transient ischemic attack

**Fig. 3:**
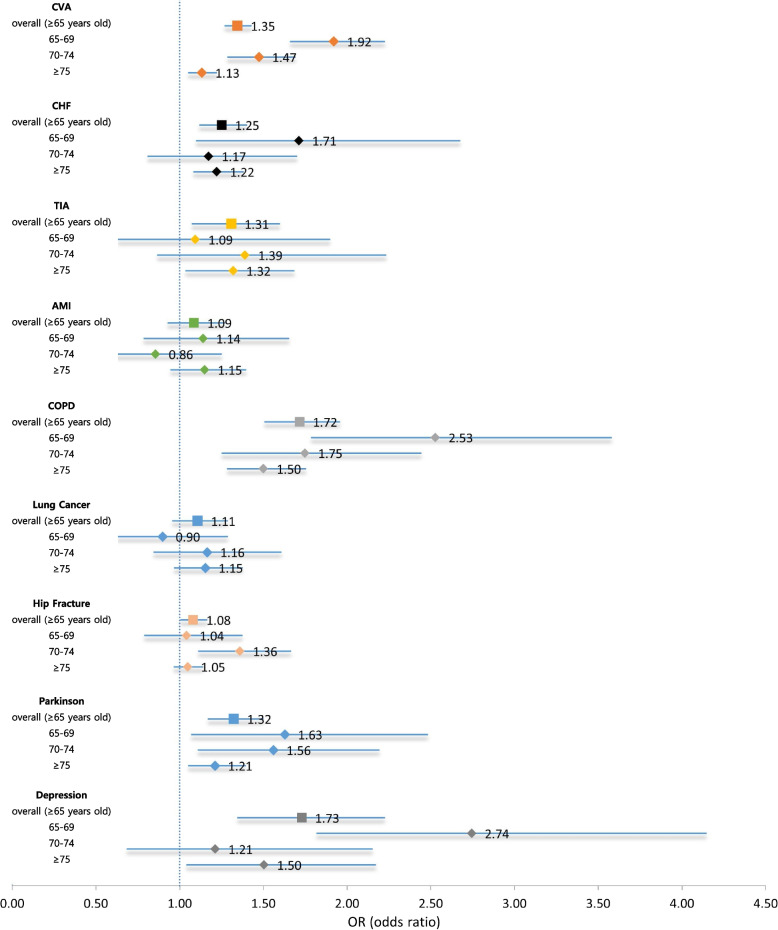
Adjusted ORs for the number of hospitalisations associated with common geriatric diseases: MA vs. NHI. *AMI: acute myocardial infarction; CHF: chronic heart failure; COPD: chronic obstructive lung disease; CVA: cardiovascular accident; OR: odds ratio; MA: medical aid; NHI: national health insurance; TIA: transient ischemic attack. *odds ratio was adjusted for age, sex, comorbidity, and area of residence using negative binomial regression models for each disease

For inpatient care, both NHI and MA groups had the largest number of hospital admissions in treating lung cancer (NHI: 1.46 admissions, MA: 1.60 admissions) and hip fracture (NHI: 1.25, MA: 1.35) (Table 2). Depression had the fewest hospital admission (NHI: 0.04, MA: 0.08). A significantly higher utilization of inpatient care measured as average annual number of hospitalizations per patient aged 65 years or above was observed among MA than NHI beneficiaries for seven diseases: depression (adjusted OR: 1.73), COPD (1.72), CVA (1.35), PD (1.32), TIA (1.31), CHF (1.25), and hip fracture (1.08) (Fig. 3). Except for TIA, lung cancer, and hip fracture, adjusted ORs decreased as patients got older for the remaining five diseases, implying that discrepancy in utilization of inpatient care between the two beneficiary groups diminished.

## Discussion

Our analysis showed that CVA was the most prevalent morbidity in Korean older adults among nine common geriatric conditions. Approximately 10% of each of the NHI (868.5 per 10,000 beneficiaries) and MA (1,197.1 per 10,000) older adults suffered from CVA. Depression has a high prevalence of 3–5% of Korean older adults (NHI: 295.1 per 10,000, MA: 481.3 per 10,000). Unlike other diseases, prevalence of depression decreased with age for MA older adults, whereas it increased for NHI older adults (Table 1). Depression is not usually accompanied by acute symptoms and relatively less urgent and severe than other diseases; hence, priority of seeking diagnosis and medical care for depression may be lower than other diseases. Especially for older adults with limited healthcare affordability, more urgent and demanding diseases may precede depression in spending money for diagnosis and treatments. Thus, our finding regarding the decreasing prevalence of depression with age for financially vulnerable groups should be interpreted with caution. The older adult suicide rate in Korea (72.0/100,000) is 3.27 times higher than the average rate of OECD countries (22.0/100,000) [16], and depression is a major risk factor for suicide [17]. Therefore, special attention and societal effort to reduce the prevalence of depression and provide more active medical intervention are necessary, particularly for economically vulnerable older adults.

We hypothesized that older adults living in extremely poor economic environments had worse health status than those from better economic classes. In relation to the common geriatric diseases, this study confirmed our hypothesis. A significantly higher prevalence was consistently observed in MA than NHI beneficiaries for seven out of nine diseases (Fig. 1), suggesting that Korean older adults living in poverty may suffer from unfavorable health disparity. Most of the nine diseases can be prevented by controlling for risk factors, such as high blood pressure and sugar, hyperlipidaemia, smoking, obesity, and low bone density, during the middle or younger ages. This implies that health disparity in the population with poor economic condition could be associated with poor management of risk factors during early years of life. In fact, the prevalence of hypertension among the lower 20% income earners of Korean adults is 30.9%, which is 4.5% higher than those in the top 20%. Hypertriglyceridemia also showed the same trend [18]. Thus, public health interventions targeting the middle aged from low economic classes to control for risk factors of major chronic diseases could be effective in reducing health disparity in the aged population.

The risk gap of experiencing geriatric diseases between MA and NHI beneficiaries was wider in younger age (65–69 years) than older age (≥ 75). For example, adjusted ORs comparing prevalence of disease among MA versus NHI beneficiaries were greater than 2.0 for COPD (2.94), hip fracture (2.72), PD (2.04), and depression (2.04) when they were 65‒69 years old (Fig. 1). Furthermore, adjusted ORs dropped (COPD: 1.67, hip fracture: 1.22) or were no longer statistically significant (PD: 1.05, depression: 1.05) when they reached above 75 years of age (Fig. 1). This finding implies that older adults in poverty start living with fragile health condition earlier and their healthy longevity, defined as years of life without illness or disability, is shorter than those with better economic statuses. For those over 75 years of age, it is thought that the physical aging mechanism has a greater effect on physical health than the health care or living environment that economic status can affect.

We examined whether there was disadvantageous discrepancy in healthcare utilization of financially vulnerable older adults. Regardless of outpatient or inpatient care, MA beneficiaries consistently tended to use more or similar healthcare resources when treating the same condition. This finding might suggest no discrimination for the poor older persons in receiving healthcare services under the universal health security system in South Korea. Higher utilization of outpatient and inpatient care by the MA could be interpreted with a different perspective: the condition of MA beneficiaries is more severe, and they require more healthcare utilization than NHI beneficiaries; further, MA beneficiaries tend to overuse healthcare resources owing to the moral hazard associated with zero or low co-payment for healthcare services. For outpatient care, the magnitude of the excess in healthcare utilization by MA beneficiaries was marginal with adjusted ORs close to 1.0, ranging from 1.09 (for depression) to 1.28 (for COPD) (Fig. 2). The excess use of inpatient care by MA beneficiaries compared with NHI beneficiaries for treating the same illness was more prominent than outpatient care: adjusted ORs for the number of hospitalizations per patient ranging from 1.25 (for CHF) to 1.73 (for depression) (Fig. 3). Greater excess use of inpatient care than outpatient care by MA beneficiaries may support our projection that the condition of MA beneficiaries was more severe than that of NHI beneficiaries when diagnosed with the same disease.

Caution should be exercised before concluding that there is no inequity in access to and provision of healthcare services for indigent older adults in South Korea based on our results of significantly higher utilization of outpatient and inpatient care among MA than NHI to treat the same diseases. As our analysis focused on the quantity of healthcare services such as the number of visits, but did not analyze quality or appropriateness of healthcare services. Assessing disparity in the quality of healthcare services requires abundant clinical information such as patient’s baseline severity, comorbid conditions, and history of treatments. Future studies using long-term individual patient data with sufficient clinical information should add more scientific evidence on healthcare disparity for indigent older adult population.

This study has several limitations. First, since HIRA-APS claims data are collected when patients were diagnosed in healthcare institutions, the prevalence based on insurance claim records can be underestimated. However, most conditions included in this study are serious and critical illness that all patients cannot avoid visiting hospitals. Thus, the prevalence of those diseases estimated from claim records was considered to be close to the actual prevalence. Second, this study classified the economic status of the patients into only two groups: NHI and MA beneficiaries. Although there is little variation in economic condition among MA beneficiaries, a wide variation of NHI beneficiaries is expected. Unfortunately, owing to the lack of information about the financial status of individual patients from HIRA-APS data, we were not able to classify the participants into more subgroups. Third, healthcare utilization included in the analysis was insurance-covered services only. We were not able to determine whether there was inequal utilization of uncovered services between MA and NHI beneficiaries. Forth, the data used in this study are from 2017 and are not the most recent. However, there were no major policy changes targeting the older adult population that could affect healthcare utilization from 2017 to the present, and the dataset was the most recent with the variables necessary for analysis. Lastly, as with all other cross-sectional studies, there are inherent limitations. Since the causal relationship is difficult to establish in cross-sectional study, only the correlation between economic status and geriatric diseases can be confirmed. Further study through a longitudinal data set will be needed to confirm the causal relationship.

## Conclusion

The higher prevalence of the common geriatric diseases in MA than NHI beneficiaries suggests unfavorable health disparity for poor older adults in South Korea. Thus, government efforts to improve the standard of living in later years of life may improve not only material well-being, but also health-related quality of life. Similar or higher healthcare utilization in treating the same conditions among MA than NHI beneficiaries was consistently observed for most geriatric diseases included. This finding suggests the possibility of little inequity due to poor economic status of the older adults in South Korea, for access to and provision of healthcare services covered by universal health insurance. Future extension of our study to the assessment of utilization of non-insurance covered services and the quality of healthcare services would offer more a concrete conclusion.

## Data Availability

The dataset used and analyzed during the current study are not publicly available due to our IRB policy, but are available from the author AYK(pharmay@yonsei.ac.kr) upon reasonable request.

## Abbreviations

AMI: Acute myocardial infarction
aOR: Adjusted Odds Ratio
CHF: Chronic heart failure
COPD: Chronic obstructive pulmonary disease
CVA: Cerebrovascular accident
HIRA-APS: Health Insurance Review and Assessment Service-Aged Patient Sample data
MA: Medical Aid
NHI: National Health Insurance
OECD: Organization for Economic Co-operation and Development
PD: Parkinson’s disease
TIA: Transient ischemic attack
